# Efficient Lazy Theta* Path Planning over a Sparse Grid to Explore Large 3D Volumes with a Multirotor UAV

**DOI:** 10.3390/s19010174

**Published:** 2019-01-05

**Authors:** Margarida Faria, Ricardo Marín, Marija Popović, Ivan Maza, Antidio Viguria

**Affiliations:** 1 Center for Advanced Aerospace Technologies, Calle Wilbur y Orville Wright, 19, 41300 La Rinconada, Sevilla, Spain; rmarin@catec.aero (R.M.); aviguria@catec.aero (A.V.); 2 Autonomous Systems Lab., ETH Zurich, 8092 Zürich, Switzerland; mpopovic@ethz.ch; 3 Robotics, Vision and Control Group, University of Seville, Avda. de los Descubrimientos s/n, 41092 Sevilla, Spain; imaza@us.es

**Keywords:** path planning, UAV, autonomous exploration, sparse grids, Lazy Theta*

## Abstract

Exploring large, unknown, and unstructured environments is challenging for Unmanned Aerial Vehicles (UAVs), but they are valuable tools to inspect large structures safely and efficiently. The Lazy Theta* path-planning algorithm is revisited and adapted to generate paths fast enough to be used in real time and outdoors in large 3D scenarios. In real unknown scenarios, a given minimum safety distance to the nearest obstacle or unknown space should be observed, increasing the associated obstacle detection queries, and creating a bottleneck in the path-planning algorithm. We have reduced the dimension of the problem by considering geometrical properties to speed up these computations. On the other hand, we have also applied a non-regular grid representation of the world to increase the performance of the path-planning algorithm. In particular, a sparse resolution grid in the form of an octree is used, organizing the measurements spatially, merging voxels when they are of the same state. Additionally, the number of neighbors is trimmed to match the sparse tree to reduce the number of obstacle detection queries. The development methodology adopted was Test-Driven Development (TDD) and the outcome was evaluated in real outdoors flights with a multirotor UAV. In the results, the performance shows over 90 percent decrease in overall path generation computation time. Furthermore, our approach scales well with the safety distance increases.

## 1. Introduction

The role of Unmanned Aerial Vehicles (UAVs) as tools in human activities only now begins to unfold. Many multirotor UAV platforms are now commercially available. Their applicability is bounded chiefly by creativity and novelty. Examples of tasks aided by UAVs are package transportation [1,2], industrial inspection [3,4,5,6,7,8], scene reconstruction [9,10,11], and environmental monitoring [12,13,14]. These applications, in daily life, take place outside controlled environments. To fully exploit the potential of UAVs, a key challenge is to plan paths and create maps in complex, unstructured environments. Resources aboard UAVs are scarce. Complex algorithms require processing power. Weight constraints limit available range of usable processors. The resources must be shared among all tasks. Despite these constraints, exploration has recently been extended to three-dimensional domains. Examples are sewer exploration [5], bridge inspection [4], and forest exploration [15].

Autonomous exploration can be formulated as an active-learning problem. It is a problem that incorporates simultaneous localization, mapping, and planning [16]. Rapid technological developments in aerial robotics and robotic sensors have motivated significant research in motion planning, as discussed in the survey of Goerzen et al. [17]. This paper focuses on the planning task in time-invariant, static, and near-static environments. These algorithms avoid the need for complex, time-consuming manual mission planning on densely occupied space.

In this research, design choices and solutions are guided by the use case targeted: a rotary wing UAV whose task is the autonomous exploration of a large, unknown, and unstructured environment. Examples of such scenarios include search and rescue, archaeological structures, and inspection of industrial facilities.

As noted by Goerzen et al. [17], the characteristics of the particular application are key in determining how to solve the motion planning problem. In an exploration scenario, the unexplored space can contain obstacles. To keep the UAV safely inside the known free space and avoid obstacles, the planner conservatively treats the unknown space as an obstacle. Another characteristic of the use case is that the map is constructed during exploration. As a result, each path request evolves over different versions of the map, the benefits of precalculated distances cannot be fully exploited. Because the UAV is omnidirectional, its orientation is not part of the configuration space. Due to the large dimensions of the space to be explored, the computation time must scale well with the length of the generated path. To be a self-contained, flexible tool, the planner must be real-time and onboard. As a result, the UAV can operate under severe restrictions of ground station connectivity and throughput rate. Tools either for industrial or commercial use must provide guarantees about the results and need to be certified. Repeatability is a vital characteristic. These traits are straightforward to achieve with deterministic algorithms. However, their major drawback is the tendency to employ more calculations, thereby limiting computational efficiency.

This research improves the efficiency of the implementation of Lazy Theta* previously presented in [7], an algorithm that has been applied successfully in competitions with multirotor UAVs [18] and fulfills the requirements mentioned above. For realistic obstacle avoidance, the concept of a flight corridor was introduced to detect obstacles that are around the trajectory. The dimensions of the flight corridor reflect the volume of the UAV and its operational restrictions, including localization uncertainty, trajectory following error, mapping errors, etc. However, this shift introduces a significant bottleneck. Obstacle avoidance now uses at least ninety-five percent of the computational time as shown later. A sparse resolution grid in the form of an octree is used to represent the world, organizing the measurements spatially, merging voxels when they are of the same state. Furthermore, this representation is well suited to store information about large scenarios as it requires little memory.

The main contributions of the paper are (1) presenting an any-angle path-planning algorithm that generates three-dimensional paths with an obstacle-free flight corridor around the trajectory, implemented over sparse grids; (2) introducing a two-phased approach to obstacle detection that qualifies Lazy Theta* for real-time, onboard usage as both a local and global planner; (3) refine neighborhood generation by taking into account the multi-resolution nature of the octree; (4) adoption of the methodology TDD paired with smart monkey testing.

The algorithm can generate paths as a local planner because the resolution of the octomap is fine enough to navigate around the obstacle. The paths created can be used directly by the autopilot. The planner is also able to generate paths as a global planner because it scales well to long paths. The path can go up to a hundred times the size of the resolution, overcoming local minima.

The implementation of Lazy Theta* as well as the maps used in testing are open sourced and available at https://github.com/margaridaCF/FlyingOctomap_code. Videos illustrating the results can be found at https://sites.google.com/view/lazythetastaronline/videos.

In Section 2 is an overview of the state of the art. The contributions are analyzed in depth in Section 3. The proposed method is described in Section 4 and tested in experimental scenarios in Section 5. Finally, the main conclusions are drawn in Section 6.

## 2. Related Work

This section reviews the state of the art in path planning, mentioning autonomous exploration. In particular, the following discussion of prior studies focuses on collision avoidance and scalability, as these are some of the critical features of the proposed algorithm.

In any robot navigation scenario, a crucial task is to, given a set of global destination waypoints, plan collision-free paths that satisfy motion constraints. Yang et al. [19] presents a thorough survey of the state of the art in three-dimensional motion planning. Within their taxonomy, algorithms are distinguished as being (i) deterministic or (ii) non-deterministic. Unlike deterministic methods, non-deterministic strategies are not guaranteed to produce the same outputs over multiple runs. As such, deterministic algorithms naturally satisfy any repeatability requirements, which commonly arise in industrial inspection scenarios.

Non-deterministic, sampling-based algorithms apply continuous path-planning to high-dimensional spaces. Some examples include Rapidly Exploring Random Trees (RRTs) [20,21] and the Probabilistic Road Map (PRM) [22]. These approaches leverage uniform sampling to grow a connectivity structure, e.g., a tree or graph, towards unexplored areas of the problem instance. More recent studies in this field [4,23,24] propose variations of RRTs to improve the optimality and computational speed of sampling-based algorithms. For UAVs in particular, Oleynikova et al. [25] and Lin and Saripalli [26] present probabilistic approaches to generate collision-free trajectories in cluttered environments. In inspection scenarios, various non-deterministic planners have emerged to sample efficiently promising viewpoint configurations in continuous space. Typically, these methods either only consider greedy next-best views [4] or incorporate a non-myopic look-ahead to escape local minima [11]. Song and Jo [27] employ a two-step strategy based on primal and dual sampling to generate paths for constructing accurate three-dimensional models of an unknown environment. The works Bircher et al. [4], Papachristos et al. [5], and Papachristos et al. [6] adopt a receding horizon planning strategy, sampling possible future configurations in a geometric random tree. More recently, Witting et al. [28] presented a polynomial trajectory-based planner which exploits a history graph to direct the growth of an RRT towards unexplored regions of a target environment. Recent work in this field by Papachristos et al. [6], and Francis et al. [29] has tackled incorporating the UAV’s pose uncertainty into the planning objective for improved map quality.

In a similar problem setup, Heng et al. [9] tackle visual exploration and coverage by performing optimization in the UAV state space. The search is done in four dimensions (position and orientation) accelerating the computation by relying on a precomputation of the swath of the motion and the sensor range shape. This approach delivers dynamically feasible plans and demonstrates scalability to office-size environments. Whereas stochastic approaches enable efficient exploration of large volumes, they cannot deliver repeatable results. The scale of the scenarios targeted in these studies varies widely. In simulated environments, e.g., [4,9,28], the workspace volumes span 1000–10,000 m${}^{3}$, whereas in experimental settings, e.g., [5,28], they are constrained to 100–200 m${}^{3}$. In contrast, the proposed approach was applied in significantly larger simulated and real environments, on the orders of 520,000 m${}^{3}$ and 11,000 m${}^{3}$, respectively.

Visibility graphs are another option for world representation. In general, the cost of building a fully connected graph presents a good trade-off when the same graph is queried multiple times. For example, Scholer et al. [30] construct a three-dimensional visibility graph for UAVs, composed of one obstacle, and two supporting graphs. The approach relies on a fully known environment. Consequently, the graph is built once, with the high build cost restricted to initialization. This requirement is incompatible with dynamic maps that are built in real time during a mission.

To enable consistent, repeatable results in large-scale environments, the strategy of this research opts for a deterministic, complete method to navigate to the map’s frontiers. One algorithm with these characteristics is A* [31], and it also guarantees to find the best path. Radmanesh et al. [32] compare various path planners in three different scenarios. Among the deterministic path-planning algorithms without associated error, A* has the smallest computation time. However, one major drawback is that paths are formed by the edges of a discrete grid. As such, their results do not necessarily correspond to shortest paths in continuous space. Often the paths have sharp edges that are difficult to track with practical controllers. The any-angle family of algorithms addresses this issue by generating paths outside the grid’s edges. The Field D* algorithm [33] uses interpolation to choose in what point of the grid’s edge to cross to the next voxel. It has been used in two dimensions for the Mars rovers Spirit, Opportunity, and Curiosity. In [34] a simulated micro-UAV vehicle goes from start to goal using an AD* search algorithm for replanning. The underlying world representation is a three-dimensional occupancy grid that is sampled where the samples are arranged as a multi-dimensional lattice.

Another any-angle algorithm is Theta* [35]. Here the path is found by evaluating the connection not only between neighbors but also between the neighbor of a candidate and its previous waypoint. Theta* is less suited for real-time constraints because of the high number of line-of-sight checks it performs [36]. The Lazy Theta* extension was presented by Nash et al. [36] and has been extensively used for two-dimensional paths generated over regular grids of various shapes [37,38,39]. This extension reduces the number of line-of-sight checks, which is a crucial aspect in alleviating the effect of the obstacle detection bottleneck. Some work has been done applying Lazy Theta* to three dimensions but always over regular grids. One example is [40], although video games are the primary use case it can be used to any continuous terrain. Garcia et al. [41] also applies Lazy Theta* for three-dimensional path planning for UAVs navigating in hazardous weather conditions. In this work are quantitatively compared A*, Theta* and Lazy Theta*. The computation time is similar, the cost is lower for Lazy Theta* as well as the number of line-of-sight checks performed. However, the time constraints only make Lazy Theta* suitable to act as a global planner. Faria et al. [7] applies Lazy Theta* to three dimensions using a sparse tree. In this type of structure, voxels with the same state are merged. The spatial clustering enables the spatial analysis to be done in a computationally efficient manner because each time a voxel is examined the corresponding volume is analyzed at once. Moreover, this permits scaling path generation to larger scenarios, e.g., a building or oil rig.

## 3. Increasing the Efficiency of Lazy Theta* for Exploration

In the adopted setup, the information from the world comes from a distance sensor mounted on the UAV. First, the distance measurements form a point cloud that translates the space around the robot. Then the point clouds are combined in the internal representation of the world, the octree. Finally, the octree merges voxels of the same value into larger voxels. However, the Lazy Theta* path planner generates a path over a graph. Let the voxel’s centers in the trees be the nodes of the graph, and the connections between neighbors the edges.

Sparse trees present an opportunity to simplify the regular grid, adjusting the resolution to the terrain configuration. Octrees are one way to represent sparse grids, they are trees with a maximum of eight children and, as a result, have a flatter hierarchy than binary trees making them faster to traverse. Both because of the focus on low memory requirements and on information organization, the octree implementation used for world representation is the octomap framework [42,43]. In the octomap implementation, information is added not only for detected occupied locations but also for the free space. The free space is extrapolated from the location of the sensor and of the obstacle. Furthermore, the spatial clustering more efficient calculations as each time a voxel is examined, the corresponding volume is analyzed at once.

The major benefits of Lazy Theta* add to the potential of the sparse tree. The any-angle property of the resulting path and the reduced amount of obstacle detection checks combined, address the computational restrictions imposed by the onboard and real-time requirements. The paths generated are smooth enough to dispense post-processing algorithms. In addition, the reduced number of calculations enable the planner to be used both at the local and the global level.

This work maintains the guarantees given in the original Lazy Theta* algorithm [36]. Excluding time restrictions, it is a complete, deterministic algorithm.

### 3.1. Flight Corridor

In Nash et al. [36] is presented the Lazy Theta* path-planning algorithm, later in Faria et al. [7] it is implemented over an octree. However, in [7] the UAV is treated as a single point. Both the UAV’s volume and the maximum distance to obstacles need to be taken into account to ensure reliable obstacle avoidance. The safety margin provides a failsafe from the different sources of error: the combined sensor error, the quantization error, and the trajectory tracking error. Additionally, to be able to fly, allowances must be made to include the human safety pilot’s reaction time in the loop.

However, an obstacle detection bottleneck arises when switching from a point vehicle type of problem (as defined in [17]) into checking the volume of the flight corridor, as shown later. A significant contribution of this work is to include realistic obstacle avoidance while keeping the runtime appropriate for real-time and onboard use.

### 3.2. Sparse Neighbors

The motivation behind selecting a multi-resolution octree grid as the world representation is to merge voxels with an equal state (free, occupied, or unknown). One consequence of this choice is the variable number of neighbors. As the number of merged voxels increases, the number of neighbors increases exponentially. At the base of the tree resolution, a voxel with the size of the resolution has six neighbors. Going up three levels, a voxel that is only four times the size of the map’s resolution can already have 384 neighbors. At the top of the tree, at the sixteenth level, a voxel can have 6,442,450,944 neighbors. The total number of neighbors depends on the configuration of the space. However, this variability in voxel size can also be used to speed up the calculations as all the neighbors with the same state are analyzed at the same time.

In [7], the neighbors are generated always assuming maximum resolution neighborhood. While this may occur, it is an extreme edge case, especially for larger voxels. An edge case is a case where among all the input variables one occurs at an extreme (maximum or minimum), even though within limits. The neighbors must be calculated in a flexible way to adapt to their variable size and exploit the multi-resolution structure of the tree. Fewer neighbors will lead to fewer obstacle checks, hence reducing the time needed for these verifications.

This optimization raises again the problem of voxel identification discussed in [7]. The two pieces of information associated with a voxel (its coordinates and its key) are not unique. Coordinates overlap as each leaf voxel in enclosed in larger parents and the key is unique only within the tree level. Fortunately, the two characteristics are unique when combined: the coordinates of the center of the leaf voxel and the voxel’s size. Combining characteristics that are not unique individually creates a composite key, a common technique in relational databases.

### 3.3. Efficient Geometric Obstacle Detection

In [7], the UAV is abstracted as a point. Nevertheless, a maximum safety margin to an obstacle must be observed all around the vehicle. In this work, the volume around the trajectory that must be free to fly is referred to as the flight corridor. The original pseudocode of Lazy Theta* [36] is not affected by the inclusion of this concept. What must change is how visibility is calculated both from a node to its neighbors, lines 12, 37 and 38 of Lazy Theta* pseudocode in [36] and between the two nodes start and end, line 35 of Lazy Theta* pseudocode in [36]. For this task, the resolution of the map is considered to be the discretization step within the flight corridor.

Let ${}^{G}S=\left(x_{S},y_{S},z_{S}\right)$ be the start position and ${}^{G}E=\left(x_{E},y_{E},z_{E}\right)$ the end position. Furthermore, let us define d as the vector that goes from the start position to the end position, $\left(x_{E}-x_{S},y_{E}-y_{S},z_{E}-z_{S}\right)=\left(d_{1},d_{2},d_{3}\right)$. Craig’s notation in [44] is adopted in this paper.

Two approaches to obstacle detection are considered in this work. First, in Section 3.3.1, the three-dimensional discretization is summarized. Secondly, in Section 3.3.2, is described the two-dimensional discretization.

#### 3.3.1. Three-Dimensional Discretization

One option for obstacle checking within the flight corridor is to segment the space in all three axes, x, y, and z. The discretization step matches the resolution of the octomap to guarantee that all voxels between the start and end positions are covered. This discretization creates a rectangular corridor, the width of the corridor is twice that of the safety margin. In the center of the corridor is d, the length of the corridor is |d|. The line segments obtained with the discretization have the direction of d and are independent of the alignment of the octomap.

#### 3.3.2. Geometrical Two-Dimensional Discretization

The flight corridor can also be represented as a cylinder with the d vector in the center and height |d| The discs in each end can be expressed as circles. Where the circle around ${}^{G}S$ is

$${}^{G}C_{S}=\left\{\begin{array}{c} {\left(x-x_{S}\right)}^{2}+{\left(y-y_{S}\right)}^{2}+{\left(z-z_{S}\right)}^{2}=r^{2}\\ d_{1}\left(x-x_{S}\right)+d_{2}\left(y-y_{S}\right)+d_{3}\left(z-z_{S}\right)=0 \end{array}\right.$$

and ${}^{G}C_{E}$ is analogous around ${}^{G}E$. Their radius is half the width of the flight corridor. Around the end position, the free space must also be verified forwards, forming the hemisphere at the end of the cylinder ${}^{G}H_{E}$

$${}^{G}H_{E}=\left\{\begin{array}{c} {\left(x-x_{E}\right)}^{2}+{\left(y-y_{E}\right)}^{2}+{\left(z-z_{E}\right)}^{2}=r^{2},\\ d_{1}\left(x-x_{E}\right)+d_{2}\left(y-y_{E}\right)+d_{3}\left(z-z_{E}\right)⩾0 \end{array}\right.$$

The flight corridor is the union of the cylinder and the hemisphere.

**Figure 1:**
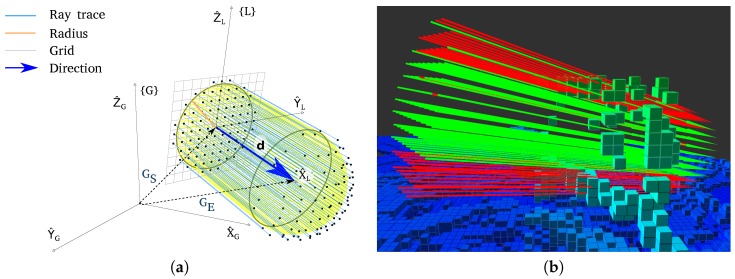
Ray traces used in the geometrical approach. The cylindrical shape with the hemisphere around the goal can be identified in both images. (**a**) Visualization of the flight corridor with the grid illustrating the space discretization resolution. (**b**) Simulation example in which red rays have collided with obstacles but green rays are in free space.

As only the bases of the cylinder are discretized, only the y and z axis need to be discretized reducing the number of discretized dimensions. The rays are cast from the base that contains the start position, ${}^{G}C_{S}$, to the hemisphere around the endpoint, ${}^{G}H_{E}$. Figure 1 shows this concept graphically both geometrically and with a simulated example.

Let us define *r* as half the flight corridor width, Δ is the map resolution and the maximum, max, is equal to 2r/Δ. Then we find the two sets of points that correspond to ${}^{G}C_{S}$ and ${}^{G}H_{E}$. We start by generating $C_{S}$ and $H_{E}$ in local frames, around the zero as

$${}^{L}C_{Si,j}={\left(\begin{array}{ccc} 0 & y_{i,j} & z_{i,j} \end{array}\right)}^{T}{,}^{L}H_{Ei,j}={\left(\begin{array}{ccc} \sqrt{r^{2}-{y_{i,j}}^{2}-{z_{i,j}}^{2}} & y_{i,j} & z_{i,j} \end{array}\right)}^{T},\forall i,j\in \left[0,\max\right]\cap Z,$$

where $y_{i,j}=i\Delta -r$ and $z_{i,j}=j\Delta -r$. The points are included in the set only if they fulfill the condition $y_{i,j}^{2}+z_{i,j}^{2}\leq r^{2}$. The number of points around the start ${}^{L}C_{Si,j}$ and the end ${}^{L}H_{Ei,j}$, as well as their relative position, can be calculated only once at the beginning. These sets of points will be referenced here as the offsets.

During the path generation, when a start-end pair is known, the transformation of these sets of points from the local frames to the global frame is composed by a rotation and a translation. The rotation is the same for both start and end and is computed as it is shown in Algorithm 1. The rotation aligns the circle and the hemisphere to be orthogonal to d. The translations, center the sets of points in the global frame as

GCS1=GLRGS0001LCS1

GHE1=GLRGE0001LHE1

**Algorithm 1** Generate a rotation matrix to transform from the local coordinate frame into the global coordinate frame. The z axis is used to calculate a vector orthogonal to d. The exception is to avoid precision issues with the cross-product due to collinearity. The final axis is orthogonal to both directions and the previously found axis. The vector $\hat{Z_{L}}$ is always pointing as vertical as possible. The algorithm is applied to each set of start and end positions as the rotation is the same for both local frames.
**Input:**d **Output:**${}_{G}^{L}R$ 1:  $\hat{X_{L}}=\frac{d}{\left\|d\right\|}$ 2:  **if**
$\hat{X_{L}}\cdot \hat{Z_{G}}\leq 0.9$
**then**
 3:   $\hat{Y_{L}}=\frac{\hat{Z_{G}}\times \hat{Z_{L}}}{\left\|\hat{Z_{G}}\times \hat{X_{L}}\right\|}$ 4: **else** 5:  $\hat{Y_{L}}=\frac{\hat{X_{L}}\times \hat{X_{G}}}{\left\|\hat{X_{L}}\times \hat{X_{G}}\right\|}$ 6: **end if** 7: $\hat{Z_{L}}=\hat{X_{L}}\times \hat{Y_{L}}$
 8: ${}_{G}^{L}R=\left\{\hat{X_{L}},\hat{Y_{L}},\hat{Z_{L}}\right\}$
 9: **return**
${}_{G}^{L}R$

In this application, the more convenient shapes around each base of the cylinder are a circle and a convex semi-sphere. However, this approach can be used with any shape.

## 4. Development Methodology

This paper builds upon the work presented in the article [7], which describes the implementation of Lazy Theta* using octomap as the world representation. The goal is to enable the generation of paths in an amount of time compatible with real-time requirements. A preliminary run of the original implementation in outdoor flights revealed that obstacle avoidance is the major computational bottleneck, as is shown later. The reduction of obstacle detection calculations was twofold. Firstly, due to the multi-resolution nature of the map, the large voxels generate many regular grid neighbors. Moreover, by discretizing in two dimensions instead of three the number of rays required to check a flight corridor is reduced. To realistically evaluate the incremental changes of the implementation, different techniques from software engineering were employed as described in Section 4.1.

The change in computational time is less evident in the context of path generation because obstacle detection stops as soon as an obstacle is found. To validate the impact of the two discretization approaches on computational time, the worst-case scenario was selected as the benchmark. Each time the corridor is searched, all rays are cast regardless of whether an obstacle is found.

The flight corridor provides a failsafe from different sources of error: (1) the combined sensor error (Inertial measurement unit—IMU, Global Navigation Satellite System—GNSS, Light Detection and Ranging—LIDAR); (2) the quantization error, percentage of voxel occupied by the obstacle; and (3) the trajectory tracking error resulting both from the controller and from external perturbations, such as wind gusts. In a practical outdoors setting, allowances must be made to include the safety pilot in the loop. The flight corridor must cater for the reaction time of the safety pilot. According to Loffi et al. [45], the minimal time to identify and react to another UAV in a collision trajectory is twelve seconds and a half. Identifying a collision trajectory with a static object is a considerably faster task. However, the study informs on how to narrow down the values for the flight corridor.

### 4.1. Software Engineering Considerations

To verify that the implementation achieves the intended results under all possible conditions is a non-trivial task. However, it is crucial to ensure that changes in the code still produce previously verified behaviors. This challenge is also faced in the area of software development and can be addressed with tools from that field.

In the development, testing, and data collection procedures several concepts from the software engineering field were employed. Different setups or development environments were successively applied to expose the program to the computational time restrictions incrementally. To create stable, well-tested code, the development was done in tandem with a suite of tests that verifies each required behavior. Finally, the data collection was automated whenever possible to generate thousands of paths.

The idea of bringing tools and paradigms from software engineering into robotics is increasingly common. One example is the European project RobMoSys [46] that uses flexible general-purpose modeling of systems with the Unified Modeling Language as a reference. Another example is the use of the continuous integration tool of software developments docker adopted in the ROS build farms, robotics companies [47] and even the ROS-Industrial Consortia [48].

#### 4.1.1. Development Environments and Data

The code was developed mimicking the environments used in continuous integration practices to deliver software. Three different environments or setups were used: development or Dev, Hardware in the Loop (HitL) and Flight. Each environment is defined by the processor and type of data used for the tests. They are progressed through in this order to face increasingly realistic and restrictive conditions, as can be seen in Table 1.

Table 1 describes the order each environment is progressed through as well as the corresponding setup. In both environments where data collection takes place (HitL and Flight), the processor used to generate a path is the same. Dev, the first environment, is reserved for a faster prototyping phase.

The distance sensor onboard is a two-dimensional laser. The maps are classified into three types: synthetic, experimental snapshots, and continuously generated. Synthetic refers to maps produced by integrating laser readings generated by the simulator. An experimental snapshot is a map created during a flight that corresponds to the state of the map at a particular moment. Finally, the continuously generated map changes throughout the runtime of the algorithm as the laser readings are continually integrated. This last environment closer mimics the exploration use case where the map is continuously generated in an unknown environment. The generation of each type of map is detailed in Section 5.1.

#### 4.1.2. Test-Driven Development

Testing is a crucial aspect in all software development, nevertheless in the case of the TTD methodology it is particularly important since for each requirement, a failing test is created, then the code is refined until that test no longer fails. To achieve stable code throughout the development process, the behavior of the methods is captured in tests that warn when the correct values are not calculated. In software engineering terminology they are called unit tests. Several tools exist to automate the testing process. The ROS integration of Google’s C++ unit testing framework gtest [49] is the tool selected for automated unit testing. In tests that address the path generation as a whole (instead of the smaller functions comprising it) the input consists of five variables: the map, the starting coordinates, the goal coordinates, the size of the flight corridor, and the maximum number of seconds the path planner has to find a solution.

Smart monkey testing is adopted to identify extreme input combinations independently of the bias of the developer. In Software Testing terminology, monkey testing refers to testing with random input. Smart monkey testing is a more specific form of testing where knowledge of the software is embedded into the test as well the capacity to report found problems or bugs. is adopted to identify extreme input combinations independently of the bias of the developer. The planner is integrated into an exploration architecture to generate a flight plan for each new goal location. The autonomous exploration process inspects a large scenario for two hours to collect laser data. As the world representation is built during exploration, the path planner is tested on different versions of the map. Consequently, the algorithm is applied to many combinations of the input variables although always in a synthetic map. This process exposes many edge cases each one is added as a unit test. The paths generated are used to relocate the sensor to the next sampling location, hence experimentally verified to be free of obstacles.

### 4.2. Automated Data Collection

To obtain an insight into the behavior of Lazy Theta*, it is tested under variable combinations of inputs. The variables that compose the input of the path planner are the starting position, the goal position, the map, the flight corridor width, and the processing time before declaring a path unsolvable.

There are some aspects to consider in particular. The length of the final path will be influenced by the distance between the start and the goal as well as by the distribution of obstacles in the environment. The configuration sparse grid must vary as well. The variability involved in generating an octree is tremendous. Each composition of voxel size and quantity has the potential to be an edge case. Setting up a particular configuration of voxels is extremely difficult. To produce the desired octree, it would require manipulating both the content and the order of each point cloud integrated into the map. The flight corridor width determines the number of line-of-sight checks per node. Increasing the width, while maintaining the maximum amount of time, can be critical in determining whether a path is findable. Finally, the number of seconds the algorithm is allowed to run will also influence the success rate. In this study, sixty seconds was fixed as the maximum amount of time allocated for path generation.

To gather information about such diverse factors automated testing was used. Only synthetic maps and experimental snapshots are compatible with this method of testing. In preparation, the map is generated and saved to a file. For each map, several points are identified as informative according to the map’s characteristics. The key characteristics of the points are adjacency to openings, proximity to obstacles, and variable distance to other points.

The tests adopt the following process: load the map from a file, verify preconditions and then generate the path. As a precondition, no obstacles can exist within the flight corridor width. Each test examines a combination of points that are used interchangeably as start and goal. These sets of tests are repeated to explore different values about the flight corridor width.

## 5. Results and Discussion

This section begins with a preliminary test of computation time to identify the bottleneck. Different types of maps were used: synthetic, static experimental, and continuously generated maps. The second subsection details each dataset and its generation. In the fourth subsection the isolated analysis of the different methods of obstacle detection is described. The fifth subsection gives a full relation of the findings for each type of map in the HitL environment. Finally, in the sixth subsection the results obtained in the outdoor flight experiments where the paths are generated onboard and in real time are described.

### 5.1. Preliminary Bottleneck Analyses

A preliminary evaluation of computation time reveals that obstacle avoidance is the most critical bottleneck of the algorithm. Table 2 shows which proportion of time obstacle avoidance corresponds to. The information was collected by recording the computational times of LTS_SN on HitL environment using experimental snapshots. Even with different margin values, obstacle avoidance always occupies over ninety-six percent of the total planning time.

### 5.2. Map Generation in the Different Environments

This study was conducted using three types of maps: synthetic, experimental snapshots and continuously generated maps. Detailed instructions are available at (https://sites.google.com/view/lazythetastaronline/home).

#### 5.2.1. Synthetic Map

**Figure 2:**
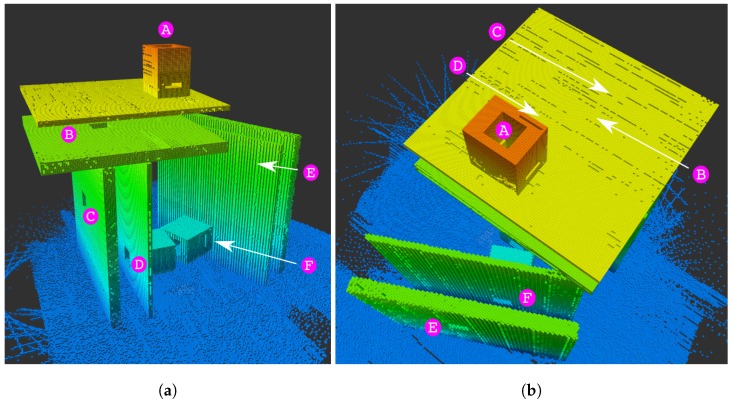
Octomap of the synthetic scenario: a three-dimensional puzzle. This synthetic map showcases the three-dimensional planning potential of Lazy Theta*. Holes in the obstacles are denoted by letters. The resolution of the map is 0.5 m such that the smaller cubes have 0.5 m edges. (**a**) Front view of the structure. (**b**) Top-down view of the structure.

The synthetic map is used in simulation and was developed to showcase the potential of the Lazy Theta* three-dimensional planning. Figure 2 shows a map of this large-scale three-dimensional puzzle. The smaller cubes have a volume of half a cubic meter, the gaps and passages are six meters wide by six meters high. The whole structure measures ninety meters by fifty-six meters and is eighty-five meters tall.

The Gazebo simulator emulated the laser measurements used to build the octomap. These measurements were generated with a configuration to match the Hokuyo 10Lx sensor mounted on the UAV platform for the flights. The PX4 simulator is used to emulate the flight of the UAV and the capture of laser measurements. PX4 is also the autopilot used in outdoor flights.

#### 5.2.2. Experimental and Continuously Generated Maps

**Figure 3:**
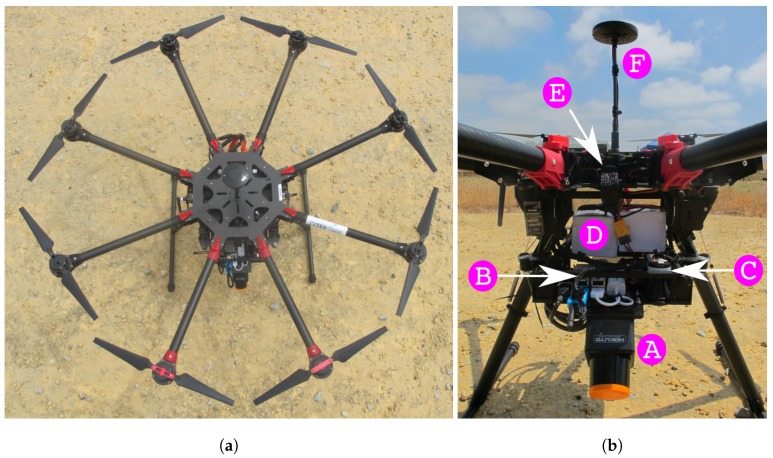
The UAV used in outdoor flights as seen from above (**a**) and from the side (**b**). The platform is a DJI S1000 and consists of one Hokuyo 10Lx sensor (A), an UpBoard with an Intel^®^ Atom^TM^ x5 (B), a 5 GHz wireless communication rocket (C), a pair of batteries (D), a Pixhawk v1 (E), a Here+ RTK (F).

**Figure 4:**
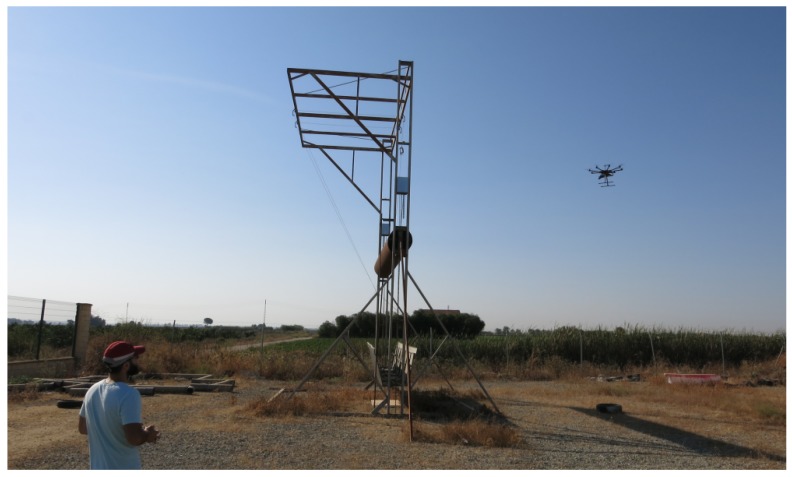
The outdoor setting for real flights using the UAV in Figure 3, the obstacle to avoid, and the safety pilot.

Both experimental and continuously generated maps are built with sensor data collected in outdoor flights. The experimental setup was a Hokuyo 10Lx LIDAR mounted on a DJI S1000 platform flying over an outdoor scenario with a large obstacle. The onboard processor was the Intel^®^ Atom^TM^ x5 on an UpBoard. The autopilot running was PX4 running inside a Pixhawk version one. Figure 3 shows the UAV and its parts, whereas Figure 4 depicts the outdoor scenario.

During the tests, the start and the goal position, the flight corridor width, and the maximum computation time were issued from the ground station. The commands passed through 5 GHz wireless communication band from the laptop to the UAV. Upon receiving the input variables, the UAV used Lazy Theta* to generate a path. As soon as Lazy Theta* reached a solution, the UAV sent the path back to the ground station through the same channel.

During a mission, the UAV continuously integrates the laser measurements into the map, even during the path generation. The path is generated directly over the continuously changing octree to avoid the additional memory needed to create a static copy of the tree. In the target use case of exploration of large environments, memory shortages could arise from duplicating the map. For the HitL environment, the map is a snapshot taken from a particular point in the execution. The snapshots enable an extensive analysis of realistic conditions.

### 5.3. Obstacle Avoidance Approaches

**Figure 5:**
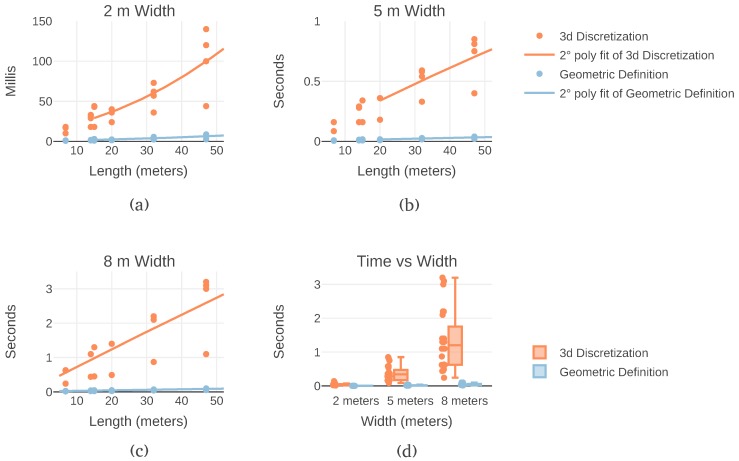
Obstacle avoidance times for varying flight corridor lengths and widths from experimental data with 0.5 m resolution. Avoidance time measures the processing time needed to cast all the rays that cover a flight corridor. Different flight corridor lengths (distance between start and end locations) and widths (safety margin) are shown. (**a**) The flight corridor measures two meters in width. (**b**) The flight corridor measures five meters in width. (**c**) The flight corridor measures eight meters in width. (**d**) Presentation of the results in the same scale to illustrate the magnitude of the variation in computation time of the two approaches and the influence of the flight corridor width.

As previously shown, obstacle detection is the most significant bottleneck in this implementation of Lazy Theta*. To directly measure the impact of the discretization some aspects are considered. First, the position of the obstacle determines how many rays are needed to find it. Furthermore, the detection stops as soon as an obstacle is found, making the computational time of generating a path dependent on the configuration of the terrain. To remove this source of variation, all the rays in the flight corridor are cast. Second, each flight corridor is sampled a hundred times for each version to remove the influence of memory caching. Caching is the mechanism of storing program instructions and data in closer, faster memory levels. Finally, in the test two input variables are sampled: the path length and the flight corridor width. Paths varied from six to forty-seven meters long. The flight corridor widths evaluated were: two, five, and eight meters. The results are shown in Figure 5a–c. In a total, a hundred and ninety-two combinations of input were evaluated. The processing time was measured both with the three and the two-dimensional discretization approaches, the results are summarized in Figure 5d.

Among all, the three-dimensional discretization always takes longer on the same path. The total time for each path generation records that geometrical detection always takes at most half the time of the three-dimensional discretization. Figure 5d shows all the measurements to the same scale. This graph illustrates how the computation time scales both regarding the length and the width of the flight corridor. It becomes apparent that the width has a much more significant impact on the computation time than the length, especially for the three-dimensional discretization.

### 5.4. Lazy Theta*

Three versions of Lazy Theta* were analyzed to evaluate the impact of each contribution on the computation time. Firstly, LTS version detects obstacles with a three-dimensional discretization of the flight corridor, the closest to the previous implementation presented in [7]. LTS_SN refers to the version that reduces the number of neighbors generated but still relies on three-dimensional discretization for obstacle detection. LTS_G version includes both the reduction of neighbors and two-dimensional discretization.

**Figure 6:**
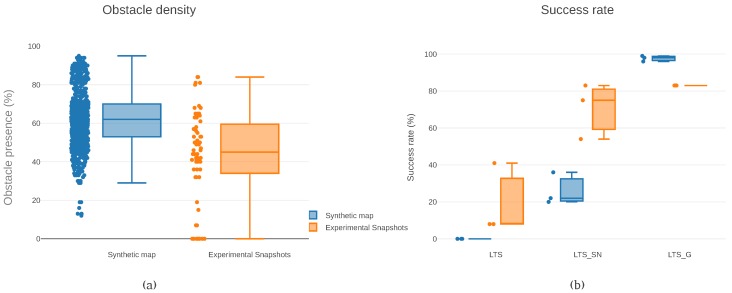
In the synthetic map, the paths generated have a maximum length of a hundred and twenty meters. In the experimental snapshots, the paths have at most thirty-three meters. Both maps have a half a meter resolution. (**a**) The obstacle density in each map. It registers the relationship between the total number of ray casts and the number of ray casts that detected an obstacle. Unknown space is treated as an obstacle. In each environment, all the individual cases used for data collection are considered. (**b**) The success rate across versions and maps types. LTS_G has the highest success rate, but LTS_SN already shows improvements in comparison to LTS. Each dot represents the combined success rate of a flight corridor width. The widths sampled were three and nine tenths of a meter, five meters, and five and four tenths of a meter.

The diversity of obstacle densities used in the samples is also plotted in Figure 6a. It is clear that a wide variety of densities is inspected in both maps, with the three-dimensional puzzle containing more obstacles, overall. The median of the obstacle density across paths in the maps recorded from experimental data is forty-five percent. In other words, every time a flight corridor was searched for obstacles, an obstacle was found in forty-five percent of the rays casted.

Figure 6b compares the success rates for the variants with different algorithms, map representation, and flight corridor widths. This figure highlights that the LTS_G version achieved higher success rates than the other versions. Figure 6b illustrates the results presented in Table 3. To note that the success rate is higher in the synthetic map (ninety-eight percent median) than in the experimental snapshots (eighty-three percent median). Interestingly, using the LTS_G version, the success rate appears to be independent of the width of the flight corridor.

The following sections focus separately on the synthetic map, experimental snapshots, and outdoor flights.

#### 5.4.1. Synthetic Map

**Figure 7:**
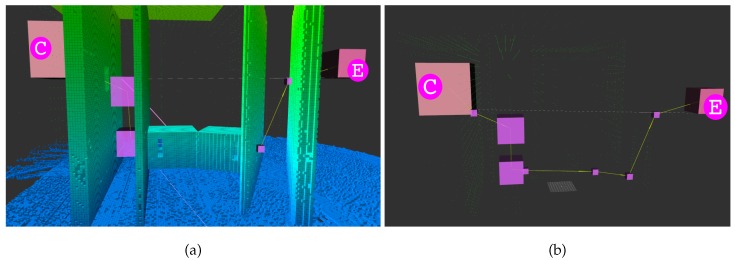
Example path (yellow lines) between two points that are 82 m apart. Purple cubes are voxels whose centers are used as waypoints, and the size of each cube reflects the size of its corresponding voxel. Adequate solution candidates are shown as small green spheres. The white line connects the start (C) and goal (E) positions. The flight corridor is 3.9 m wide, the map’s resolution is 0.5 m, the gaps and passages are 6 m wide by 6 m tall, and the whole structure measures 90 m by 56 m and is 85 m tall. (**a**) The image shows both the generated path and the world representation. (**b**) The same path is shown without the occlusion of the world representation. Additionally, the centers of the voxels candidates adequate for the solution are plotted as small green spheres.

Figure 7 shows an example of a path generated by Lazy Theta*. The path was generated with the LTS_G version. Qualitatively, the figure illustrates the three-dimensional capabilities of the algorithm. Lazy Theta* can generate a path that passes through a complex series of openings. The letters have the same correspondence as seen in Figure 2. In the different perspectives is more explicit that the openings are not aligned along any of the three axes. Throughout all the path the desired flight corridor width is observed, acting as a safety margin.

**Figure 8:**
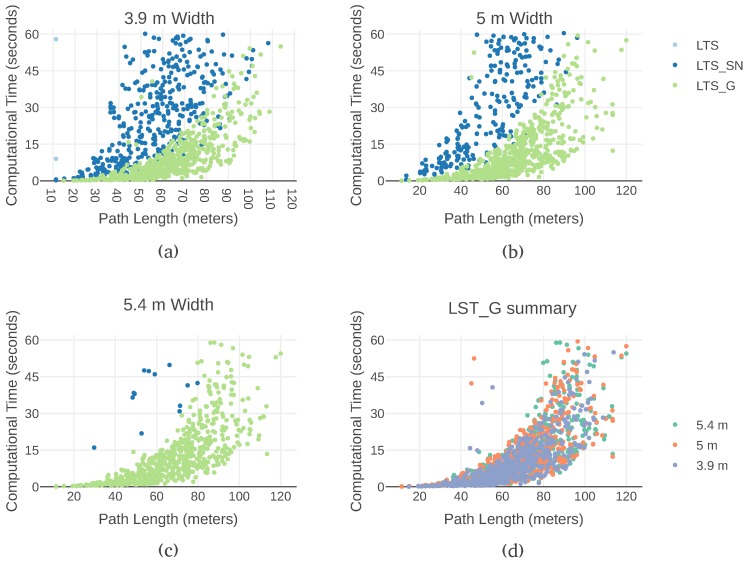
Computational times needed to find paths using three variants of Lazy Theta* in the simulated puzzle for different flight corridor lengths and widths. The points selected as start and goal were used interchangeably. The computation time need to generate paths that keep a free flight corridor was measure for: (**a**) a width of 3.9 m; (**b**) a width of 5 m; and (**c**) a width of 5.4 m; (**d**) The results obtained with LTS_G with the three flight corridor widths.

To show how the three versions fare across a broad range of input sets, Figure 8 portrays the computation time of various paths with different flight corridor widths. Each of the first three plots is associated with a particular corridor width, with the data points grouped by version. In all the graphs the points create the shape of an upward bent cone. The cone has a wider or narrower base depending on the number of ray casts involved. This shape is explained by the influence of the variability of the configuration of the map on the computation time. As paths grow longer, there is more space for variability, which results in high variability of computation time for a given path length.

The LTS version is only able to generate a path twice and only for the smaller flight corridor width. In Figure 8a, two lighter blue data points are present for over ten-meter paths. In Figure 8b,c, no data point from this version is present at all. LTS_G is always faster than LTS and LTS_SN, independently of the width or the length of the flight corridor. This result validates the hypothesis that discretizing the flight corridor in two dimensions reduces processing time. In LTS_SN, all three dimensions are discretized and sampled separately. In LTS_G, the points to cast the rays for the flight corridor are calculated in different coordinate frames. This approach allows for the discretization to be performed only for the width and the height but not the length. Each ray cast traverses the flight corridor from the start circle to the farthest point of the end hemisphere.

In all versions, a broader corridor has less successfully generated paths and the paths that are generated take longer. This results from the increased number of queries to the map. The discretization step is the same, but a larger corridor needs to be covered. On the one hand, more queries require more time, on the other hand, a larger corridor allows for a larger number of possible map configurations.

For the LTS_SN version, the effect of the increased number of raycasts in wider corridors is clear. Narrower corridors allow paths between greater distances to be found. With a width of three and nine tenths meters paths around a hundred and ten meters are established. Whereas with a five-meter corridor width, the length of the path does not reach a hundred meters. And with a five and four tenths meter width, the longest path found is eighty meters.

Figure 8d illustrates the effect of the variability of the map. In this graph, only the data points from LTS_G are plotted. The shapes of the upward bent cone of each corridor overlap each other. However, the cone is wider for the five and four tenths meter corridor then for a cone with a width of three and nine tenths of a meter. A broader cone on the horizontal axis is intuitive, as longer paths take more time to generate. A wider cone in the vertical axis reflects variability in the computation time for the same path length. The variability displayed is easily explained, the computation time is highly dependent on the underlying configuration of the octomap. As depicted in Figure 1, the flight corridor volume is covered by multiple rays. The maximum number of rays is the product of the octomap resolution and the flight corridor width. However, the search ends as soon as an obstacle is found. Therefore, in Figure 1, the number of rays present corresponds to the worst-case scenario for the obstacle search. Outside this maximum, how many rays are cast depends entirely on the location of the obstacle within the flight corridor.

#### 5.4.2. Experimental Snapshots

**Figure 9:**
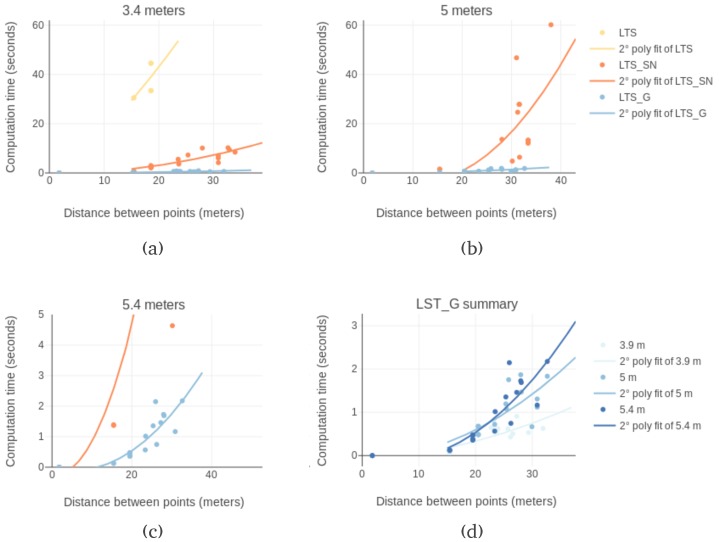
Computation times for different experimental snapshot flight corridors. The same paths are generated with increasing flight corridor widths. (**a**) Paths calculated with a 3.4 m flight corridor width. (**b**) Paths generated with a 5 m flight corridor width. (**c**) Paths computed with a 5.4 m flight corridor width. (**d**) The impact of the width of the flight corridor for LTS_G.

The computational time of the different Lazy Theta* versions over the experimental snapshots is depicted in Figure 9. In this setting, the original algorithm (LTS) can only find a significant number of feasible paths with a flight corridor width of three meters and nine tenths. In the cases a path is found, it takes much longer compared to either of the other two versions. LTS_SN can handle flight corridors of the same width but as the flight corridor widens, the path generation starts to fail. In successful cases, the computation time increases dramatically with the path length. LTS_G is a lot less sensitive to the width of the corridor. In Figure 9d, the influence of the corridor width is compared. Despite the increase in computation time, it is always under three seconds, well below the set sixty-second limit.

#### 5.4.3. Outdoor Flights with a Rotary Wing UAV

**Figure 10:**
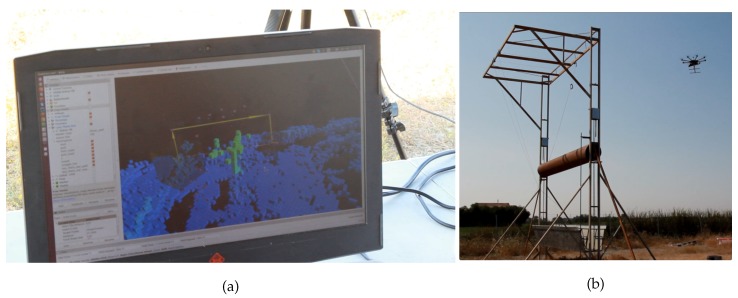
Photographs from outdoor flights. The UAV avoids an obstacle autonomously (**b**) that appears green in the map (**a**) that is generated in real time. In (**a**), the small cubes’ edges are 0.5 m.

Results using the LTS approach are not shown because no feasible paths could be generated in this environment. Figure 10a shows the resulting path received from the UAV using the LTS_SN version to avoid the obstacle depicted in Figure 10b. Here, it is clear that the solution not only avoids the obstacle but also maintains a safety distance from it along the path.

**Figure 11:**
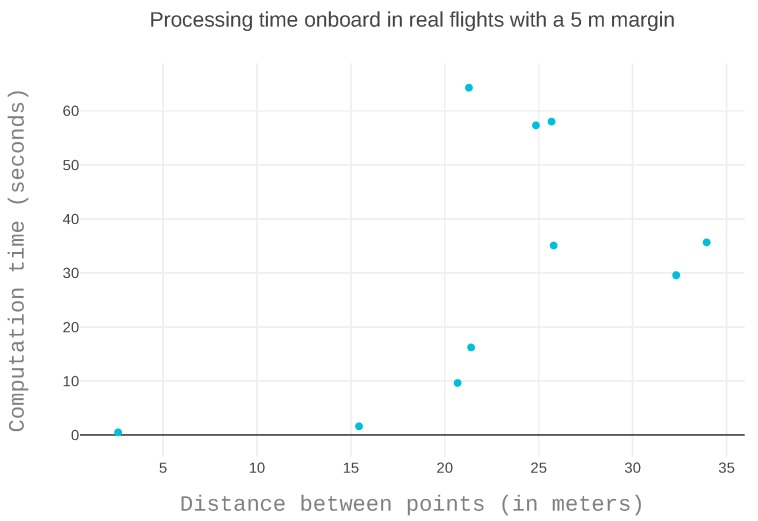
The computation time of the paths generated in real time, onboard during flight.

The data points are insufficient to outline a shape in Figure 11. However, as we have previously analyzed a three-dimensional puzzle in simulation, we can see that the points also fit the shape of an upward bent cone. The small distance takes very little time to solve. The variability in time increases with the length of the path. The twenty-meter mark is representative of the impact of map configuration on paths of the same length. For the same path length, the path generation time varies from ten to over sixty seconds. It is reasonable to assume that more data would follow this trend.

## 6. Conclusions and Future Work

This work sets out to qualify Lazy Theta* presented in [7] as a tool in the context of autonomous exploration of large scenarios. Consequently, the path generation must be done in real time and onboard, further restricting the computation time. Moreover, the path must always be at a minimum distance to occupied or unknown space. The associated calculations make obstacle detection the most significant bottleneck.

To generate paths a hundred times longer than the map resolution within the time frame two optimizations are introduced. On the one hand, the voxels composing the neighborhood of another voxel are calculated taking into account the sparse grid that represents the world. In LTS_SN the success rate increases to consistently over eighty percent. On the other hand, obstacle detection calculations are reduced by restricting the space discretization of the flight corridor to two dimensions and bringing the success rate to over ninety percent. Additionally, the software development methodology adopted is TDD paired with smart monkey testing.

Lazy Theta* can generate paths both as a local and a global planner. At the local level, the resolution of the octomap is fine enough to navigate around the obstacle. The paths created can be used directly by the autopilot. Lazy Theta* can also plan for longer paths at the global planning level. Because of the any-angle characteristics of Lazy Theta*, the path is smooth enough to avoid a post-processing smoothing algorithm, in the context of exploration.

With LTS_G, the two-phase, geometrical approach to obstacle detection allows the shape of the flight corridor to be decoupled from its position. The shape can be calculated only once at initialization, and the position is calculated for each collision check. This highly effective strategy can be applied to many other bottlenecks. Future work should focus on using it to calculate the information gain of trajectories and points.

The contributions keep the deterministic nature of Lazy Theta*. One set of input variables will always generate the same path. Because the outcome is repeatable, the path planner is uniquely suited for applications that need to be certified.

In the future, the final solution will be further tested outdoors and integrated into an autonomous exploration architecture. The two-part, geometrical method to analyze space can be used in many ways: to evaluate the information gain of frontiers, to embed information gain into the heuristics of Lazy Theta* or even to calculate the observation points of the frontiers according to the sensor.

## Figures and Tables

**Table 1:** Listing of three development environments. Environments are defined by processor choice, the type of map used, and the data collection location.

Order	Development Environment	Type of map	Processors	Data Collection
1	Dev	Synthetic and experimental snapshots	Intel^®^ Core^TM^ i7	-
2	HitL	Synthetic and experimental snapshots	Intel^®^ Atom^TM^ x5	Yes
3	Flight	Continuously generated	Intel^®^ Atom^TM^ x5	Yes

**Table 2:** Preliminary inspection of the bottleneck. In this table are shown the computation times involved in generating a path. Firstly, the total time used to find a path is shown. The total time can be decomposed in time spent on obstacle detection and the remaining time. The second column details the computational time needed to detect obstacles, both in milliseconds and as a percentage of the total time. In all flight corridor widths, obstacle detection comprises over 96% of the entire time, exposing the bottleneck.

Total Computation Time for	Total Computation Time Used in Obstacle Detection		Flight Corridor Width
Path Generation (Milliseconds)	(Milliseconds)	(Percentage)	(Meters)
5,809,367	5,627,883	96.9%	2
18,211,941	17,698,871	97.2%	2.5
37,417,587	36,723,194	98.1%	3

**Table 3:** The amount of path generation attempts and the amount that succeeded, for each version, in each map.

	Synthetic			Experimental Snapshots		
	LTS	LTS_SN	LTS_G	LTS	LTS_SN	LTS_G
Path requests	1.118	2.396	2.021	72	72	72
Successful requests	3	700	1.984	14	51	60

## Abbreviations

The following abbreviations are used in this manuscript:

GNSS	Global Navigation Satellite System
HitL	Hardware in the Loop
IMU	Inertial measurement unit
LIDAR	Light Detection and Ranging
LTS	Lazy Theta* offline version
LTS_SN	Lazy Theta* Sparse Neighbors
LTS_G	Lazy Theta* Sparse Neighbors and Geometrical two-dimensional discretization
RRT	Rapidly Exploring Random Trees
PRM	Probabilistic Road Map
TDD	Test-Driven Development
UAV	Unmanned Aerial Vehicle
